# High-throughput characterization of the ovarian cancer HLA peptidome

**DOI:** 10.1186/2051-1426-2-S3-P139

**Published:** 2014-11-06

**Authors:** Andrea Patterson, Wilfried Bardet, Curtis McMurtrey, Saghar Kaabinejadian, Ken Jackson, William Hildebrand

**Affiliations:** 1 University of Oklahoma Health Sciences Center, Oklahoma City, OK, USA

## Background

T cells recognize cancer cells via human leukocyte antigen (HLA)/peptide complexes, and class I HLA-restricted tumor infiltrating T lymphocytes correspond to favorable prognosis in several cancers, including ovarian cancer--the most deadly gynecologic disease [1]. The accurate identification of tumor-specific HLA/peptide complexes is pivotal to their exploitation as targets in immunotherapies such as peptide vaccination, transgenic T cell therapy, or antibody tumor targeting. Here, a high-throughput peptide analysis method was used to directly sequence the HLA-A*02:01 peptidome of ovarian cancer cells and to identify HLA/peptide complexes distinct to tumor cells.

## Methods

A secreted form of HLA-A*02:01 was stably transfected into the SKOV-3, A2780, and OV-90 human ovarian cancer cell lines, as well as the immortalized normal ovarian epithelial cell line FHIOSE. Yields of HLA in excess of 10 mg were harvested from bioreactors of each cell line and purified from supernatants by affinity chromatography. The bound peptides were purified from the HLA and fractionated by two dimensions of high-pressure liquid chromatography followed by mass spectrometry for peptide sequencing.

## Results

For each cell line, approximately 14,000 non-redundant peptides were identified at a 1% false discovery rate against a decoy database. A clear overarching HLA-A*02:01 motif was observed. Upon comparison, >100 peptides appeared absent from the line derived from normal ovarian epithelium but were consistent to all three ovarian cancer lines. In addition, 41 peptides derived from the tumor antigen human epidermal growth factor receptor 2 (HER2) were sequenced from the HER2 overexpressor SKOV3. Eight of these ligands confirm predicted peptides from the literature that have shown immunogenicity, including the highly targeted E75.

## Conclusion

Class I HLA gathered from tumor cells provides for the direct characterization of the cancerous peptidome. Ovarian cancers are highly heterogeneous, but this high-throughput peptide sequencing method is able to detect and highlight the consistencies in cancer-associated HLA peptide presentation--at least among three disparate cases. It has also provided the first direct sequencing evidence of HER2 peptides, confirming eight predictions and revealing 33 novel tumor ligands from this highly targeted cancer protein. In characterizing and comparing cancerous and non-cancerous peptidomes, this study has highlighted multiple candidate peptides for tumor-specific targeting in ovarian cancer immunotherapy.
